# Quantitative EEG Features of Level of Consciousness in Critically Ill Nonagenarians

**DOI:** 10.1093/geroni/igaa057.401

**Published:** 2020-12-16

**Authors:** Shawniqua Williams Roberson, Kevin Haas

**Affiliations:** Vanderbilt University Medical Center, Nashville, Tennessee, United States

## Abstract

The standard for monitoring sedation levels in critically ill patients is intermittent bedside evaluation, and is prone to anchoring bias. Quantitative electroencephalography (qEEG) allows automated processing of recorded brain electrical activity and could be used to continuously monitor level of consciousness in critically ill patients. The majority of qEEG studies have included persons 80 years of age or less, and the qEEG profiles of nonagenarians have been incompletely characterized. Knowledge of the qEEG patterns of patients 90 years and older is essential for appropriate interpretation of such metrics in this population. This retrospective cohort study characterized qEEG profiles of acutely ill nonagenarians. We investigated whether the relationship between qEEG and level of consciousness differed between patients with and without a history of dementia. We included patients 90-100 years old admitted to Vanderbilt University Medical Center who underwent EEG and as part of their clinical care. We compared qEEG features to nursing-defined level of arousal as measured by the Richmond Agitation-Sedation Scale (RASS) in patients with and without history of dementia. Between January and December 2019, 26 nonagenarians underwent EEG for clinical purposes. One study was excluded due to excessive artifact. Of the remaining, 6 (24%) were male and 18 (72%) were Caucasian. Among all patients, RASS decreased with increases in EEG theta variability (coefficient -7.7, 95%CI -10.6 to -4.8). This relationship was not significantly modified by history of dementia (coefficient of interaction term -0.36, 95%CI -3.7 to 2.9). Dementia does not impact qEEG features of level of consciousness in nonagenarians.

